# Multifocal Choroiditis and Panuveitis - difficulties in diagnosis and treatment

**Published:** 2017

**Authors:** Neacșu Gheorghe Boricean, Oana Roxana Scripcă

**Affiliations:** * Ophthalmology Department, Emergency County Hospital, Brașov, Romania

**Keywords:** Multifocal Choroiditis and Panuveitis, HLA A29, HLA B27, Aripriprazole, OCT, Fluorescein Angiography

## Abstract

We present the case of a 49-year-old patient who was treated with Aripriprazole in context of Paranoid Schizophrenia. The patient had a history of numerous Panuveitis recurrences for the left eye, which led to a marked decrease of the vision VA-NLP and was diagnosed with Multifocal Choroiditis and Panuveitis for the right eye. The examination revealed VA of 20/ 200 for right eye, keratic precipitates, and vitritis. Fundus aspect of the right eye showed multiple discrete, ovoid, yellowish-grey lesions at the posterior pole and periphery, optic disc oedema was present. The Human leukocyte antigen typing of group A, ancillary investigation (OCT, Angiofluorography, B-mode ultrasonography) and fundus examination confirmed the diagnosis of Multifocal Choroiditis and Panuveitis but we did not exclude antipsychotic-related chorioretinopathy or a Birdshot-like Syndrome.

## Introduction

Modern ophthalmology has made significant progress in recent years both in terms of technical arsenal used for diagnosing, monitoring and therapeutic alternatives, which rely, for example, on laser therapy, intraocular devices or molecules obtained by genetic engineering.

However, in current practice, unfortunately, there are still complex situations that benefit more or less of these new possibilities, which dramatically affect people’s lives. 

## Case report

We present the case of S.G, a 49-year-old woman from rural area who came to our practice complaining of decreased vision, moderate photophobia, and floaters in the right eye (OD), symptoms that appeared 7 days earlier. The patient had a history of numerous Panuveitis recurrences for the left eye, from February 2014 to October 2015 (OS), which led to a marked decrease of the vision: her Visual Acuity (VA) OS was no light perception. During 2015 and 2016, the patient followed an intensive treatment with corticosteroids (Triamcinolone + Bevacizumab intravitreal, systemic and topic corticotherapy). On detailed questioning, she mentioned that she was treated with Aripriprazole in context of Paranoid Schizophrenia for 5 years. She did not know of any systemic disorders and pathologies or any heredo-collateral antecedents.

Her initial visual acuity was 4/50 (not correctable) in the right eye and no light perception in the left eye. Intraocular pressure was 8 mmHg in OD and 4 mmHg in OS. The pupillary reflexes were abnormal in both eyes, reduced in the right eye, and absent in the left eye.

The slit lamp examination of the anterior segment OD revealed conjunctival hyperemia, fine grey keratic precipitates (KP), Tyndall 1+, floaters and dense opacities. The examination for OS revealed discreet conjunctival congestion, small and round pigmentary KP, diffuse iris atrophy, pupillary fibrin membrane, and the lens could not be seen.

**Fig. 1:**
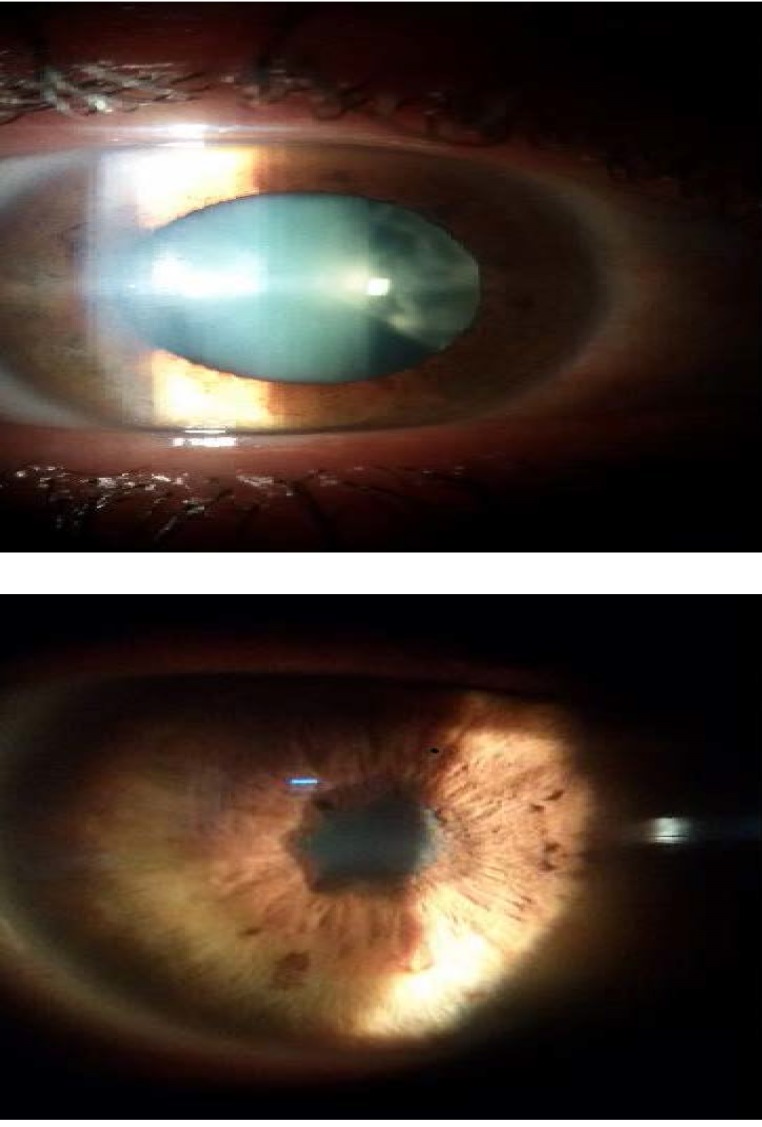
Anterior segment examination OD and OS

Fundus examination revealed optic disc oedema, flame-shaped hemorrhages, dilatation of the veins and multiple, discrete, yellowish-grey lesions in the posterior pole of the OD. The retinal periphery for OD was normal.

**Fig. 2:**
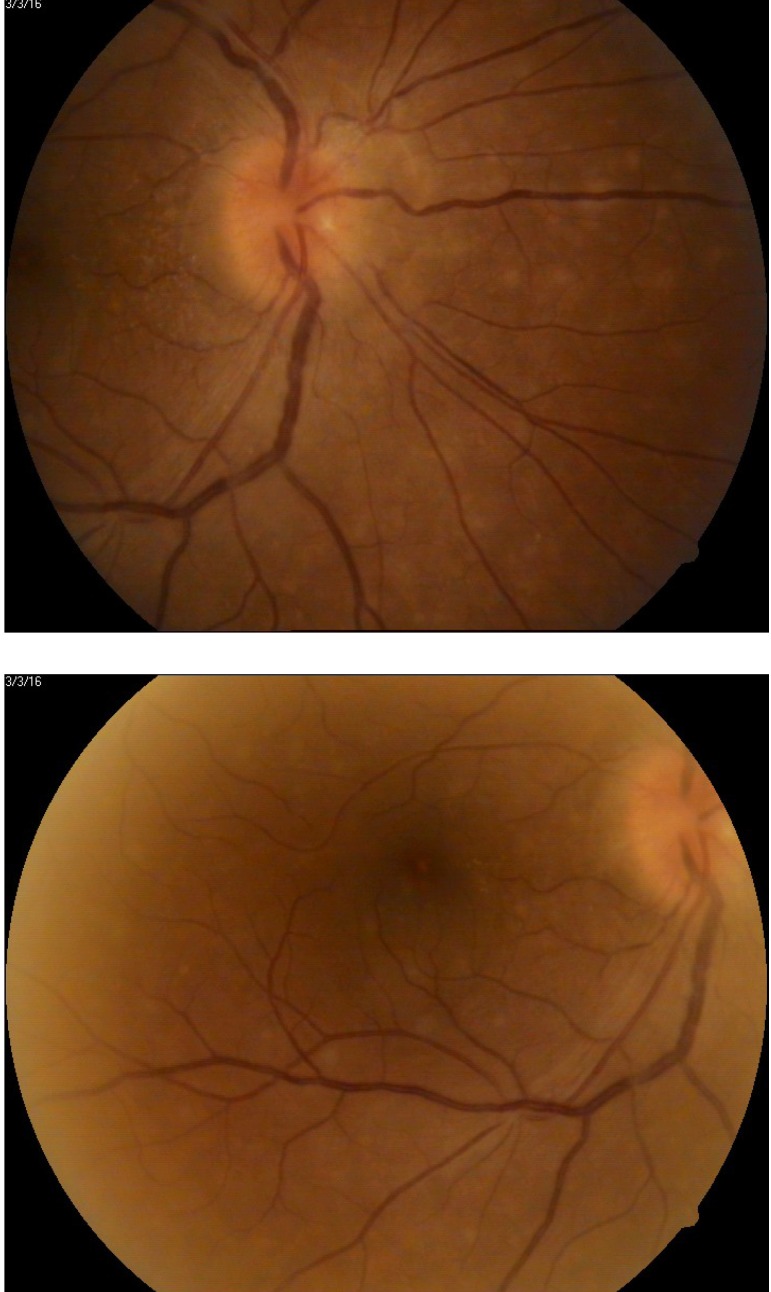
Color fundus photography OD

The B-scan ultrasonography showed no particular pathology for the OD, but for the OS, the scan revealed total serous retinal detachment.

**Fig. 3:**
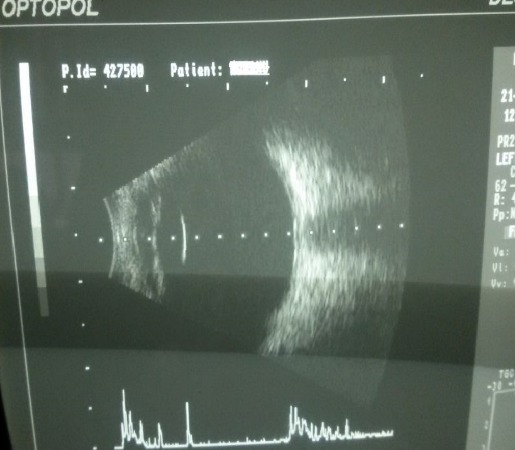
B-scan ultrasonography OD

**Fig. 4:**
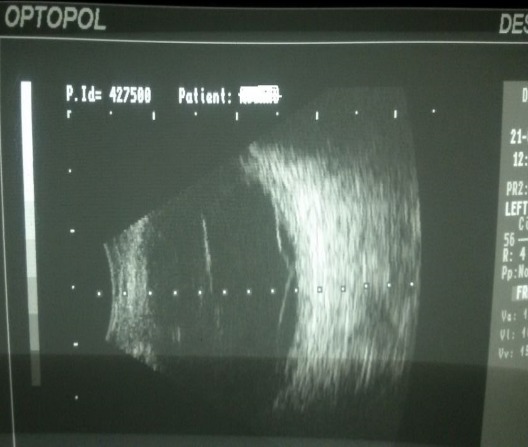
B-scan ultrasonography OS

We performed a Fluorescein Angiography (AFG) that showed multiple hypofluorescent spots disseminated at the posterior pole in the arterial phase (starting at about 11 to 12 seconds after injection of fluorescein) and especially in arteriovenous phase. After completing the passage of fluorescein (which took about 25 to 26 sec) and beginning the phases of circulation, the spots became hyperfluorescent, discreetly increasing in intensity but not extending. A gradual hyperfluorescence of described lesions and optic disc hyperfluorescence could be seen in the late stages of AFG.

**Fig. 5:**
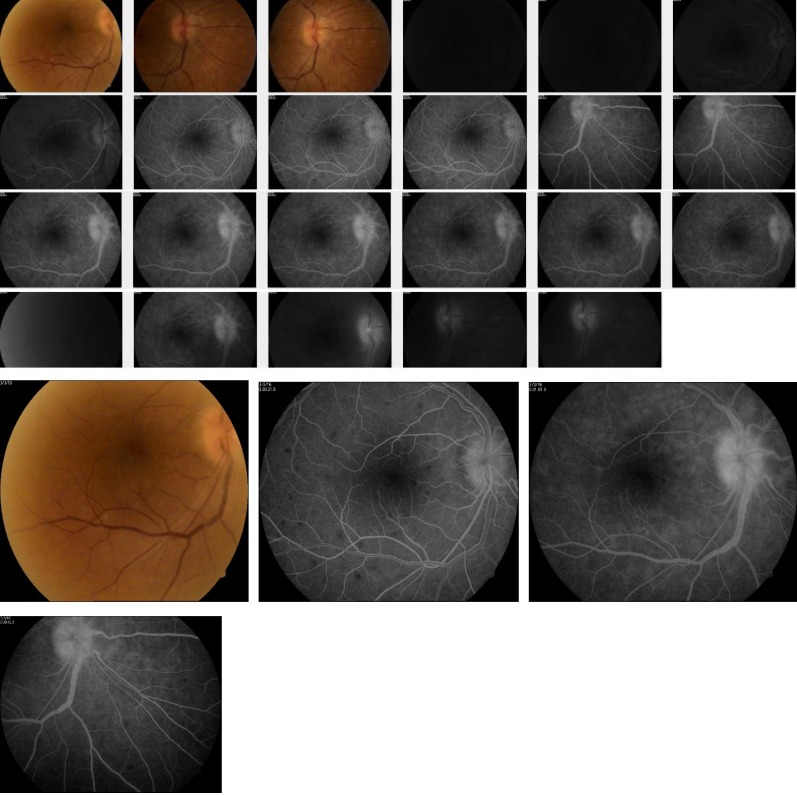
Fundus Fluorescein Angiography

We performed an ancillary testing for the diagnosis of comorbidities, including imagistic evaluation. Chest X-ray and spinal-sacroiliac joints were normal, while the head MRI showed chronic maxillary sinusitis on the right side. The common blood tests, such as CBC (complete blood count), erythrocyte sedimentation rate and inflammatory markers, were normal. We also made tests for rapid plasma reagin (RPR), TPHA, VDRL, rheumatoid factor, antinuclear antibodies, anti-double stranded DNA, C3 and C4 complement, total protein test and serum protein electrophoresis, angiotensin-converting enzyme and they all were within normal limits. We made serological tests to look for certain antibodies for CMV, Toxoplasma gondii, Toxocara canis, Herpes simplex, Epstein Barr, Rubella infection and antibodies for Lyme disease. They were also within limits.

The Quantiferon TB gold test did not detect the presence of specific gamma interferon mycoplasma tuberculosis infection.

Genetic tests were required for a differential diagnosis with Birdshot Chorioretinopathy. By a low dissolution typing method, the HLA typing A locus detected the A02 and A11 alleles, but the allele A29 was not detected. We also performed the HLA B27 typing with a negative result.

Neurological examination was within limits.

Judging the fundus aspect, we concluded that the patient was suffering from a White Dots Syndrome. The investigations and the blood test results suggested Multifocal Choroiditis and Panuveitis.

Due to the significant visual loss, we administered methylprednisolone intravenous pulse therapy for six days, 1g of methylprednisolone divided in two doses per day. This was followed by oral methylprednisolone 32 mg/ day decreasing gradually.

The condition evolved favorably under steroids treatment with a partial recovery of the vision. Upon discharge from hospital, the VA OD was 1/ 2 without correction.

After 5 weeks of low dose methylprednisolone, the patient returned to the hospital with symptoms similar to the first presentation. Her VA OD was 1/ 2 without correction.

Examination of the posterior pole revealed the yellowish-grey lesions, optic disc oedema, and a vitreoretinal traction zone in the macular area. The OCT showed hyper-reflectivity of retinal pigment epithelial layers, discontinuity between internal and external layers of the retina, vitreomacular traction and an epi-foveal membrane. The OCT also confirmed the optic disc oedema.

**Fig. 6:**
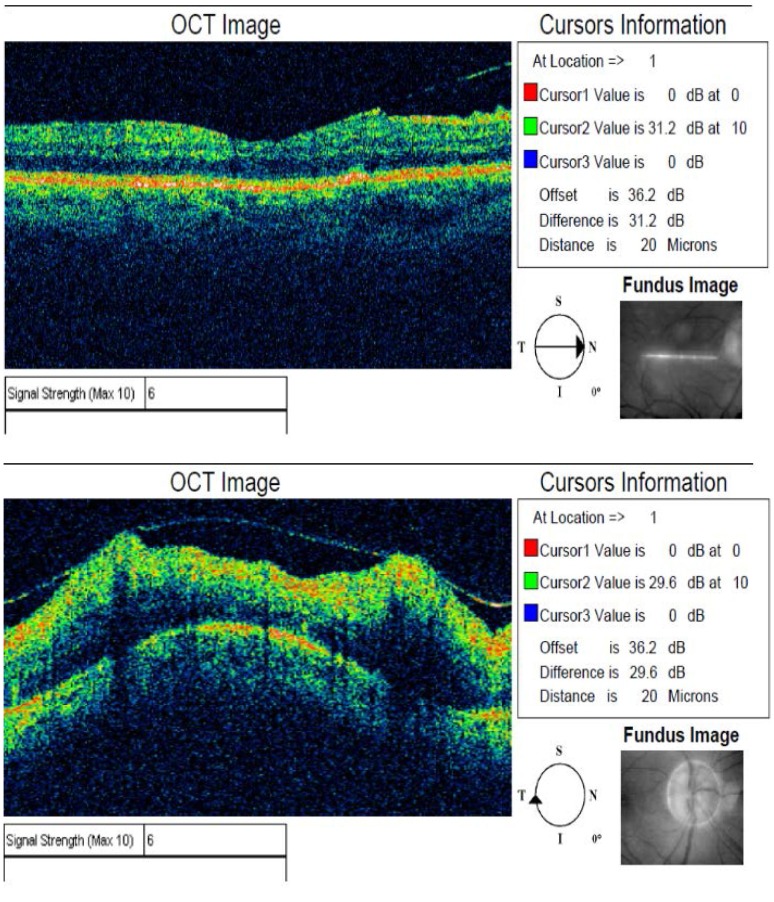
Stratus OCT profile report OD

The Perimetry suggested diffuse low light sensitivity, absolute paracentral scotoma, and relative scotoma alternating with retinal areas with normal sensitivity.

**Fig. 7:**
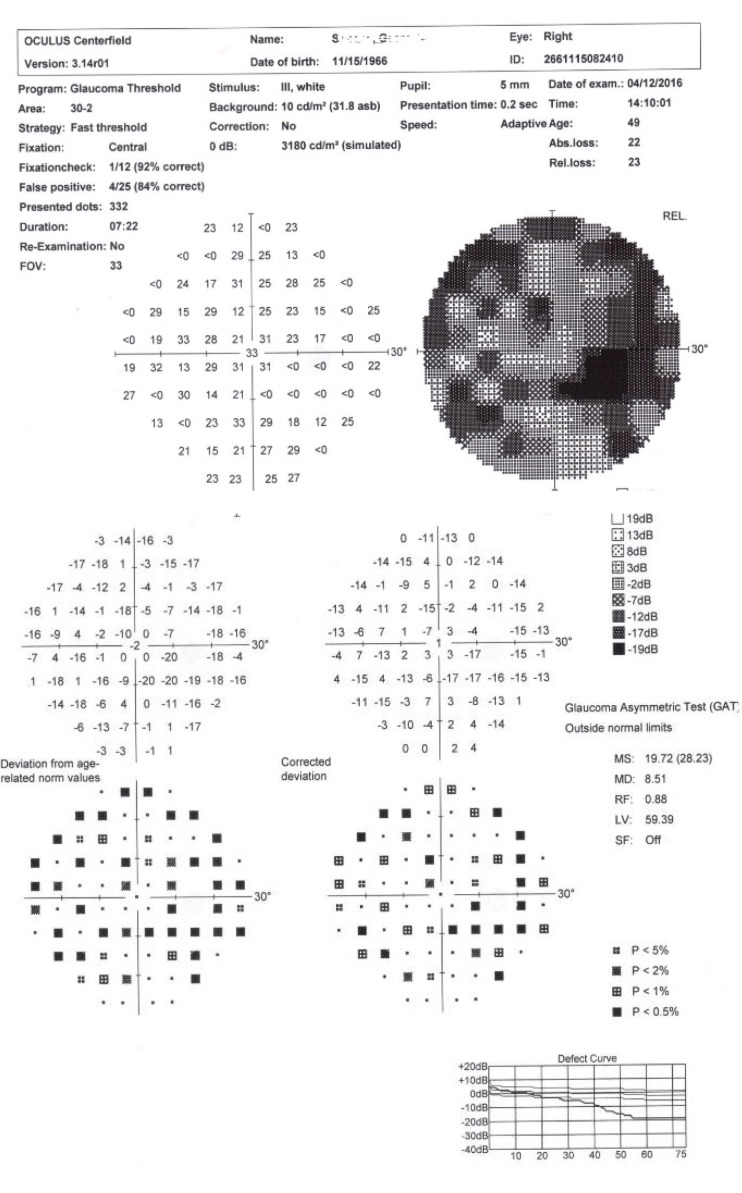
Oculus Centerfield Perimetry OD

Due to the complex situation, we asked the University of Birmingham for a second opinion. Alastair Denniston MD (Consultant Ophthalmologist-Uveitis and Medical Retina Center) also classified the case as a Multifocal Choroiditis with Panuveitis and suggested Mycophenolate immunosuppression therapy.

The patient was evaluated in the Rheumatology Department of “Sfanta Maria” Hospital in Bucharest and the immunosuppressant treatment and immunological re-evaluation were recommended.

In May 2016, the patient began immunotherapy with Methotrexate 12.5 mg in combination with a low dose of Prednisone. 

After four months of combined therapy and with a stationary evolution, without relapses, a vitrectomy via pars plana was made. Dexamethasone and Vancomycin were injected intravitreal during the intervention.

## Discussions

Multifocal Choroiditis and Panuveitis is a condition characterized by intraocular inflammation and multifocal choroidal lesions occurring in the absence of any known ocular or systemic disease. Bilateral involvement is present in approximately 66% to 79% of the patients and seems to have a predilection for females in the second to sixth decade of life [**2**]. Most patients have a variable amount of anterior segment inflammation and vitreitis. Patients report blurred vision and floaters while photopsias might be present. Acute choroidal lesions vary in size and number, from several to several hundred, are yellowish to gray in color, and located at RPE level. Older lesions appear atrophic [**1**].

Although it has been reported that patients with multifocal choroiditis and panuveitis have serologic evidence of a chronic or persistent Epstein-Barr virus infection [**3**], our patient did not seem to have stigmata of Epstein-Barr, the Anti EBV IG M, and IG G were negative.

Several diagnostics were taken into consideration and immunological determinations were made like anti-Toxoplasma antibodies that led to antiparazitary treatment for 7 days without improving vision. We performed HLA typing A locus [**4**,**9**] by a low dissolution typing method for a differential diagnosis with Birdshot Chorioretinopathy, A29 allele was not detected. The clinical criteria did not reveal Behcet disease. HLA B 27 was negative. HLA A 11 allele was positive and typical for sympathetic ophthalmia [**5**] but, having no trauma in history, the diagnostic is unlikely.

Aripriprazole is an atypical antipsychotic. It is recommended and primarily used in the treatment of schizophrenia and bipolar disorder. Retinal toxicity due to Aripriprazole was also incriminated in some medical studies [**6**] and the antipsychotic was stopped without any significant changes.

Medical treatment of uveitis is often limited and controversial causing physicians to turn to complex therapies and drug combinations in order to provide safe and effective therapies for this ocular disease [**10**].

Glucocorticoids are used as topical, periocular or systemic treatment in order to reduce anterior segment and vitreous inflammatory activity, macular detachment and oedema. Antivascular endothelial growth factor (anti-VEGF) can be used as well. 

When corticosteroid therapy does not have the desired effect, immunomodulatory agents represent the next category of medication used. This includes the broad categories of antimetabolites (azathioprine, methotrexate, and mycophenolate mofetil), alkylating agents (cyclophosphamide, chlorambucil), T-cell inhibitors (cyclosporine, tacrolimus) and cytokines (interferon alpha) [**1**,**7**,**8**].

The diagnostic for the patient’s left eye was multifocal choroiditis with intermediary uveitis. She was administered corticotherapy but the evolution was without any favorable results. Even anti-VEGF intravitreal injections were made with no positive outcome. Given the history of the left eye, the corticotherapy complications involving Cushing’s syndrome and possibility of vitritis appearance due to low Prednisone dosage, the patient is now on combined therapy: Methotrexate 12.5 mg/ week and Prednisone. 

The above presented case showed the difficulty of differential diagnosis between syndromes that fall into the category of panuveitis for a patient with antipsychotic treatment and without major changes in complex biological test and imaging examinations.

## Conclusions 

This case was a huge challenge for the above-mentioned reasons. Due to the difficulty of the differential diagnosis and the effort needed to receive the appropriate immunomodulatory therapy, the patient might get discouraged from fighting the disease.

We believe that in complex cases of panuveitis, corticosteroid-immunomodulatory combined therapy gives the best results. It is in the patient's interest to have access to modern immunomodulatory therapy in order to treat syndromes that fall in the category of panuveitis, because the lack of treatment can lead to irreversible loss of vision.

## References

[1] Yanoff M, Duker JS (2009). Ophthalmology.

[2] Saxena S, Saxena RC (2009). Retina Atlas a Global perspective.

[3] Tiedeman JS (1987). Epstein-Barr viral antibodies in multifocal choroiditis and panuveitis. Am J Ophtalmology.

[4] Kuiper J, Rothova A, de Boer J, Radstake T (2015). The immunopathogenesis of birdshot chorioretinopathy; a bird of many feathers. Prog Retin Eye Res.

[5] Reynard M, Shulman IA (1983). Histocompatibility Antigens in Sympathetic Ophthalmia. American Journal of Ophthalmology.

[6] Faure C, Audo I, Zeitz C, Letessier JB, Robert MP (2015). Aripriprazole-induced chorioretinopathy: multimodal imaging and electrophysiological features. Doc Ophthalmol.

[7] Goldberg NR, Lyu T, Moshier E, Godbold J, Jabs DA (2014 ). Success with single-agent immunosuppression for multifocal choroidopathies. Am J Ophthalmol.

[8] Okada AA (2005 ). Immunomodulatory therapy for ocular inflammatory disease: a basic manual and review of the literature. Ocul Immunol Inflamm.

[9] Denniston AK, Minos E, Barry RJ, Southworth S, Folkard A, Murray PI, Duker JS, Keane PA (2016 ). Birdshot chorioretinopathy: current knowledge and new concepts in pathophysiology, diagnosis, monitoring and treatment. Orphanet J Rare Dis.

[10] Denniston AK, Barry RJ (2015 ). Controversies in the Pharmacological Treatment of Uveitis. Curr Pharm Des.

